# Understanding the value of a forecast using an online game

**DOI:** 10.1371/journal.pone.0335212

**Published:** 2026-05-22

**Authors:** Niko Yiannakoulias, Catherine E. Slavik

**Affiliations:** 1 School of Earth, Environment and Society, McMaster University, Hamilton, Ontario, Canada; 2 Legacy for Airway Health Chair in Health Communication, Faculty of Health Sciences, Simon Fraser University, Research Scientist, Legacy for Airway Health, Vancouver Coastal Health Research Institute, Vancouver, Canada; Roma Tre University: Universita degli Studi Roma Tre, ITALY

## Abstract

This research illustrates the use of an online game to study the value of forecasts under conditions of information uncertainty. The objective of the game is for players to plant crops in field locations in a way that maximizes a payoff, with the option of paying for forecasts that help make better decisions. We found that while players fall short of theoretically perfect play, they generally make decisions that improve their score. Players both under and overpay for forecasts that reduce uncertainty, but exhibit behaviour that shows they roughly understand the expected value of forecasts. The small sample size and demographic homogeneity of the participants limit our ability to generalize these results, but the results suggest that game environments can be useful platforms for augmenting existing primary data collection methods. This is especially poignant today since with the assistance of AI, researchers with little to no programming experience can now design and develop games in ways that would have been prohibitively costly less than a decade ago.

## Introduction

Forecasts feature into everyday life; for example, in weather, financial investments, airplane arrival times, sports, and predictive algorithms for recommending movies. Many forecasts are cost-free and integrated seamlessly into routine activities, and most forecasts can directly impact decisions. For example, a weather forecast can indicate whether to wear a sweater to work, and a price forecast for a financial security can influence whether or not a person purchases it. In the classical sense, the objective value of a forecast depends on how effectively it reduces uncertainty and its potential to improve the outcomes of decisions [1]. However, the perceived value of forecasts may often deviate from their objective value and understanding these perceptions can help more effective communication across a breadth of applications.

In this research, we study the perceived value and use of forecasts using a relatively novel methodology: an online game. This game challenges players to make decisions about forecast information based on their understanding of expected value in the context of a casual game playable on Internet capable devices (mobile phones, laptops, tablets). Using online computer games as research platforms remains relatively new, but they can augment traditional experimental platforms when lower levels of participant engagement are a concern. Engaging games can motivate intrinsically rewarding play and can provide contextually rich experiences that may expand the generalizability of traditional laboratory experiments. While not a replacement of other methods, they may be a low-cost and scalable option for researchers that wish to better understand the impact of information on decision making.

The purpose of the research is twofold. First, it demonstrates the use of an online game to collect primary data in a decision-making task. The game used here does not include interactions between players that require strategic thinking; rather, through a combination of sequential decisions players make, the game reveals preferences for information and player decision making patterns. Second, the game provides some insight in how forecasts are valued, and the degree to which forecasts can be effectively used in decision making. Herein we address three research questions: 1) do players *without* forecasts behave rationally? 2) do players *with* forecasts behave rationally? and 3) how does the price of a forecasts impact decision making? The contribution to theory is modest, particularly since the method used here is relatively novel and the sample size is small and not generally representative of the population. Nevertheless, this game-based approach allows us to observe how people interact with sequential and uncertain information that would be difficult to capture with traditional survey methodologies.

## Background

### Research gaming experiments

Research gaming involves using games and features of game design as instruments to collect data from research participants. This distinguishes them from scholarly applications that use games to for other non-entertainment tasks. Serious games, for example, are most often used to describe games for education, training and learning [2]. Advergames are used in marketing and sales [3]. Gaming is also used to explore how AI and machine learning can be used to solve real world problems [4]. However, unlike these applications, research games are used to collect data from participants involved in game play in which the game is a model of some real-world framework for decision making [5]. In this way, research games can be used as platforms for testing hypotheses, exploring social systems and modelling behaviour and decision making.

Game-like experiments have been part of the social sciences for decades. In experimental economics, games have been often used to simulate markets and auctions [6–8] and in a variety of coordination games for which theoretical analysis proves challenging [9]. This frequently involves experiments in which players, interacting directly or indirectly with each other, make decisions based on the rules and payoffs of the experimental platform, often with an incentive structure that is a direct function of game performance. In social psychology and political science, games have been used to understand systems of social behaviour, including leadership [10] and collective behaviours [11]. The platforms for conducting these experiments includes interactive laboratory exercises, board games and more recently, computer games that can be played on mobile devices.

Recent examples have shown that online gaming has proved feasible as a multi-device platform for running research experiments [12,13]. In one recent example, researchers used an online multiplayer game to empirically test theoretical models of cooperation and resource governance, showing how game-based platforms can successfully capture complex human behaviors that agree with theoretical predictions [14]. Computer-based research games are particularly useful for collecting complex and hierarchical and sequential response data because they provide a convenient setting for research questions that involve multiple related decisions over time. Game environments also provide a context for players to naturally connect these decisions to one-another—since players receive feedback from decisions within the flow of game play. In one example, researchers used a game to explore the decision making associated with housing and flood protection [15]. The platform had players make spending decisions that impacted the availability of resources for other spending decisions, but in a way that is common in gaming—with a running budget, and visual indications of resources available. Data were collected at all stages of the game, making it possible to analyze and deliberate over connections between decisions.

Online computer games are also highly scalable, which make it possible to collect large quantities of data per participant. Any in-game decision of a participant can be logged seamlessly into the game-design in a way that does not intrude on the experience of game play. For games that require players to make many decisions, this can result in a large quantity of data per participant. This logging of data can even include user experience data, including mouse movements, keystrokes and time between decisions.

Finally, games can immerse participants in an experience that motivates more engaged, authentic and meaningful participation in an activity [16]. Participants in a ‘flow states’ associated with game-like activities may exhibit greater focus and engagement [17], an idea supported by growing evidence that games and game elements can increase engagement in a variety of activities [18–22]. This can distinguish research games from the tedium often associated with other forms of data collection used in the social sciences in which the primary motivation is often the incentive payment rather than the activity itself.

### The value of forecasts

It has been known for some time that when information is scarce, people often deviate from classical rationality, relying instead on heuristics and avoiding uncertainty. In classical experiments decades ago, research showed that people preferred options with certain probabilities over options with unknown probabilities [23]. Other literature has revealed that people do not immediately use all available information to make decisions, but rather, accumulate information and make gradual shifts in expectations over time [24]. More recent experiments have extended these ideas. For example, people tend to accept ‘good enough’ options rather than exhaustively searching through and comparing all options available [25]. Information also tends to exhibit a diminishing returns effect, such that new information provides smaller marginal benefit [26], and there is evidence that people value paid information over free information, resulting in beliefs that are more extreme when paid for than when free [27].

Today, forecasts of information are available to the public in many aspects of daily life, and often available for free or at a very low cost. One practical challenge of evaluating the value of a forecast lies in distinguishing between information that meaningfully decreases uncertainty and information that merely provides a sense of confidence without tangible improvement in decision quality [28]. Moreover, useful information, even when curated by experts, is not always followed [29], and instead, factors like socio-demographics, previous experiences [30], peers [31] and even weather [32] can influence decision making. To date, considerable research into the perceived value of forecasts has focused on specialized applications, such as the perception of weather and natural hazard warning systems [33–35]. Recent literature in human-environmental systems has highlighted the critical role of “knowledge feedback” in which agents receive information about changing states of a system that stabilizes decision making and prevents resource collapse [36–38]. Nevertheless, there remains a need for more empirical work that provides insight into the ways that a general audience of information consumers think about the value of forecasts.

This research uses an online game to understand the value of a forecast. The game platform is well suited to the sequential nature of first receiving forecast information and then using it to make decisions. This platform allows us to understand the value of a forecast as well as the impact of that value on the decisions that people make. The objectives are to explore the value of games as platforms for understanding the value people put on forecasts of information as well as the impact that forecasts have on decisions. The results offer some insight into how online games can be used to understand complex and sequential decision making, as well as some evidence about how forecasts are valued and used when making decisions under conditions of uncertainty.

## Methods

### Recruitment and ethics

Participants were passively recruited through social media posts (Facebook, Twitter and Reddit) and a website over a 12-month period From August 2023 to August 2024. Due to the passive recruitment from social media posts and the internet across multiple platforms, the number of individuals exposed to recruitment materials could not be reliably quantified. Participation was restricted to persons 18 years of age and older. Participants were provided a letter of information prior to participation that summarized the purpose of the research and the procedures involved in participation. Participants expressed consent by clicking a button in an online form, at which point participants were directed to a short pre-game demographic survey. After filling out the survey, they proceeded to the online game. This research was approved by the McMaster Research Ethics Board (#4823).

### Field of fortunes game dynamics

**Fig 1:**
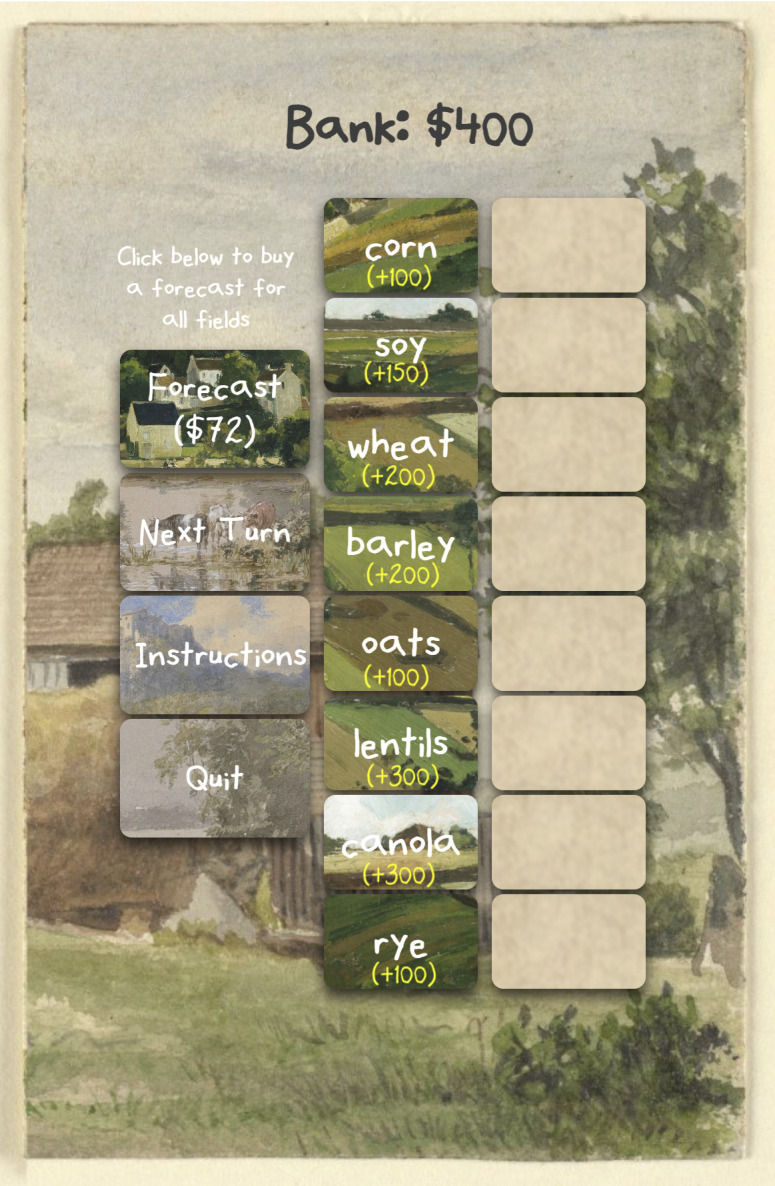
Main game display of the Field of Fortunes game. The player’s primary task goal is to maximize their bank balance (top center) by planting crops across eight available fields. Key user elements include: (Left) Action buttons to purchase a forecast for the current turn, progress to the next turn, view instructions, or quit; (Center) A list of eight crop types with their respective payout values; and (Right) Interactive field slots where crops are assigned.

The Field of Fortunes game was developed as a web app using the following tech stack: Javascript, CSS and HTML for web-client interactions, and PHP (5.x) and Mysqli for server-side operations and data storage. At the time of first playing the game, a random unique identifier was stored on a player’s device to track the identify of the player through the game, as well as repeated plays of the game. All game data were saved; however, they were not linked to the identity of the players, nor to the pre-game survey. The game was scaled to work similarly on computers and mobile devices (Fig 1). Players had the option of clicking on an instruction video with instructions on how to play at any time during the game’s operation. Participants could withdraw at any time and choose to exclude their data from the research. All anonymized data and R code are available online: https://github.com/yiannakoulias/FoFData.

During the game the player’s objective is to plant crops while trying to maximize revenue in the bank at the end of the game. The player has 8 types of crops, each with a financial return when planted: corn ($100), soy ($150), wheat ($200), barley ($200), oats ($100), lentils ($300), canola ($300) and rye ($100). There are 8 field locations where one crop can be planted with no more than one crop per field. The starting bank for the player is $400. At the start of each turn, each crop on a field has a chance of success ranging from 0 to 100%. By default, these probabilities (as percentages) of success are unknown to the player. However, at the start of every turn, the player can purchase a forecast that will tell them the probability that a crop will be successful if planted. This information is available for all field locations in the turn in which a forecast is purchased. The cost of the forecast is determined randomly for each player (values between $1 and $100, uniform distribution) at the start of the game and remains the same for every turn and every subsequent game play. Every turn, each type of crop can be planted on up to one field. If a crop is planted successfully, the player gets its value added to their bank, and the crop is no longer available for planting in the game. If the player plants a crop on a field and the crop fails, then the player loses money equal to one half the value of the failed crop. Every turn the player also pays $100 in operating costs. Once all eight crops are planted or the player has a negative bank total, the game is over, and the player has a score equal to the money in the bank.

An example sequence of game play is as follows. Turn 1, the player does not pay for a forecast. They plant corn and lentils. Corn fails, but lentils is successfully planted. At the end of the turn the balance is calculated as: $400 (starting funds) - $50 (1/2 the value of corn) - $100 (operating costs) + $300 (lentils). Total = $550. On turn 2, the player pays $75 for a forecast. They plant barley, canola and wheat. The barley and wheat succeed, but canola fails. At the end of the turn the balance is calculated as: $550 (balance from previous turn) - $150 (1/2 the value of canola) - $100 (operating costs) - $75 (forecast cost) + $200 (barley) + $200 (wheat) = $625. For turn 3, the player pays $75 for a forecast. The player plants canola, corn, soy, oats, rye, and all succeed. At the end of the turn the balance is calculated as $650 (balance from previous turn) - $100 (operating costs) - $75 (forecast cost) + $300 (canola) + $100 (corn) + $150 (soy) + $100 (oats) + $100 (rye) = $1225. Since all 8 crops are planted, the game is over, and the final score is $1225.

### Research question 1

Every turn, players must decide whether to buy a forecast or not, what crops to plant, and what field locations to plant them on. The crop values are known, but the probability that a crop will succeed on a field is not known unless the player buys a forecast. For a given crop planted on a given field, the expected value (EV) is


$$\begin{array}{c} EV=pV-(1-p)\frac{V}{2}, \end{array}$$ (1)

where p is the probability that the crop will be successful, and V is the value of the crop. This can be simplified to


$$\begin{array}{c} EV=V\left(\frac{3p-1}{2}\right)\text{.} \end{array}$$ (2)

Using (2), we see that any *p* > 1/3 will yield a positive expected value for a crop, and therefore a crop should be planted at any location with a p greater than 1/3. For players that buy a forecast, planting a crop on a field will depend on EV, and the rational player can discriminate—planting on some field locations and not on others. For players that do not buy a forecast, they do not know the probability that their crop will be successfully planted. Without spending money on a forecast, and absent any other information about crop success, it is reasonable for a player to assume that p = 0.5 for all field locations. Substituting this value into (2), gives an EV = V*0.25. This can be used to derive a table of returns for different values of p (Table 1).

**Table 1:** Expected values (EV) for each crop for given probability (p) of success.

	Value ($)	EV($) _p = 0.5_	EV($) _p = 0.1_	EV($) _p = 0.6667_
Corn	100	25	−35	50
Soy	150	32.5	−52.5	75
Wheat	200	50	−70	100
Barley	200	50	−70	100
Oats	100	25	−35	50
Lentils	300	75	−105	150
Canola	300	75	−105	150
Rye	100	25	−35	50

Given that all EVs are greater than 0 when p = 0.5, the rational player without forecast information about the success of their crops should attempt to plant all their crops and should continue doing so until the game is over. If a player plants all crops in the 8 field locations successfully in turn 1, they achieve the maximum possible score of $1750 ($400 starting money + $1450 from crops -$100 in operating costs) and the game ends immediately. The longer it takes to plant all field locations, the lower the score once all 8 field locations are planted, since players must pay $100 in operating costs every turn in addition to the costs of failed crops. As such, even if every planted crop succeeds, planting crops over multiple turns is less profitable than planting them all at once. This leads to our first research question:

***Research question 1: In turn 1 of a game, do players without forecasts attempt to plant crops in all 8 available field locations?***

### Research question 2

Players can buy a forecast to inform themselves about the specific probability of success for crops planted in available field locations. With this information, rational players should choose to plant crops on field locations in a way that ensures their greatest net revenue. The revenue maximizing strategy is to plant the most valuable crops at field locations that are most likely to be successful, and the rank correlation of the probability of success for a planting on a field and payoff size should equal 1. This is an optimal strategy since when a crop fails, half the cost of the failed crop must be paid on the next turn. If players acted rationally, we should expect them to place higher-value crops on the field locations with the greatest chance of success. When decisions are complex—because there are more field locations to consider planting crops on—players may not make full use of the information due to information overload. Moreover, the cost of information could influence player’s perceptions of value—with more expensive forecasts leading to fewer errors. This leads to our second research question:

***Research question 2: For all turns in which a player buys a forecast, do players make decisions that maximize expected value and do the cost of the forecast and the complexity of the decision impact player decisions?***

### Research question 3

Players are aware of the price of a forecast at the start of their game. The value of information per crop can be calculated as the ratio of the return from planting without a forecast and the return from planting with a forecast. With a forecast available, a player should only plant when p > 1/3, and if the player assumes a uniform distribution, this means that they should expect to plant a crop two thirds of the time. Within the range of when a player would plant a crop, p can vary between 1/3 and 1, with 2/3 as the median of forecast probabilities. Substituting the value of 2/3 into (2) yields an average return of V/2 when a forecast is used. However, since a rational player with a forecast will only plant 2/3 of their crops on average, the overall expected value of each field is 2/3*V/2, resulting in an expected V/3 (33.3%) return on crops. As shown above, the value from planting without a forecast is 25% (V/4). The value of forecast per crop is the difference between these two values, which gives the following


$$\begin{array}{c} VOF=\frac{V}{3}-\frac{V}{4}=\;\frac{V}{12}\text{.} \end{array}$$ (3)

For turn 1, the total value of a forecast (TVOF) is the sum of all crop values divided by 12 (100 + 150 + 200 + 200 + 100 + 300 + 300 + 100)/12 = $120.83. This means that at turn 1, it is rational for all players to buy a forecast, since the forecast cost in the game is bounded at $100, which is less than the TVOF. However, as the game progresses, a player should only buy a forecast when TVOF is greater than the price of the forecast. For example, if only three crops need to be planted (corn, oats and rye), then the TVOF is ($100 + $100 + $100)/12 = $25, meaning a player should only buy the forecast if it is less than $25. This leads to our third research question:

***Research question 3: How does the value of a forecast influence whether a player buys a forecast?***

### Analysis

The game data have an hierarchical structure: decisions within turns, turns within games, and games within players. This means that the analysis needs to address intra-level dependence in response data. This is a repeated measures problem, and we address it using a generalized linear mixed model (GLMM) and the lme4 R library [39]. In all cases the dependent variable is a dichotomous categorical variable representing a binary player decision. The specific structure of the model depends on research question, but the general framework is to understand fixed model effects as features of the game conditions, and the random effects as latent player/game/turn specific factors that explain the decisions players make that are not explained by the fixed effects included in the model.

Players have access to instructions that explain the operation of the game. There is some evidence that instructions are less useful for simple games than trial and error and exploration [40]. Nevertheless, seeking instructions may reveal some curiosity about the game, a higher level of engagement and an impulse to do well. It may also impact the decisions players make; with more information, players seem likely to perform better on all decision tasks. For this reason, seeking instructions is included as a main fixed effect in all models, then as an interaction term. The interaction term is retained only when the Akaike Information Criterion (AIC) with the interaction term is reduced by more than 1 when compared to the model without the interaction term.

## Results

Based on the pre-game survey, seventy-eight percent of participants were under 35 years of age, 52% self-identified as female and 77% reported having completed one or more university degrees.

Resulting game data consisted of 711 total turns within 153 unique games played by a total of 54 players. In the 711 turns played, there were 1636 crop planting decisions made by players. Participants played the game 2.815 times on average, with 55.6% playing two or more times, and 9.3% playing eight or more times. Each game took an average of 4.647 turns, with a minimum of 1 and a maximum of 14 turns. The mean player score at the end of the game was $290, with a highest score of $1448 and a lowest score of -$425. Thirty-eight (70.37%) of the players consulted instructions at least one time.

### Research question 1

71.9% of players bought a forecast on the first turn of the game. Of the players that did not buy a forecast in turn 1, 39.5% planted all their crops in turn 1; of those players who did buy a forecast, 26.4% planted all their crops in turn 1. A two-sample test of independent proportions with a null hypothesis that population proportions are equal yields a Z-test statistic of 1.5765 (p = 0.1149). However, repeated plays means that the observations are not independent of one another, so we use the GLMM to determine if there is an association between whether all crops were planted in the first turn and whether a forecast was purchased. The random effect for this model is at the player level. Results are found in Table 2. Predicted probabilities from this model are similar to the cross-tabulated values above; of those who players that did not buy a forecast in turn 1, 39.8% planted all their crops in turn 1; of those players who did buy a forecast, 22.9% planted all their crops in turn 1. This result does suggest that players who purchased a forecast were less likely to plant all their crops. The large random intercept effect shows considerable within-respondent clustering in the choice to plant a crop; in this case, there is strong evidence that players exhibited unique individual and somewhat consistent behaviours when playing the game multiple times. To get a sense of this, we calculated the predicted probabilities of players planting all their crops given the estimated random and fixed effects, finding that for those who purchased a forecast, predicted probabilities of planting all crops ranged from ~5% to ~64% within one standard deviation of the mean. For players that did not buy a forecast, this varied from ~10% to ~80% within one standard deviation of the mean. This variability shows that the bulk of variation in player decisions is associated with unmeasured player attributes rather than the decision to buy a forecast or not. This is confirmed with inter-class correlation coefficients; 43.2% of the variance on the latent (log-odds/logit) scale is attributable to between-player differences in this model when the fixed effects are held constant [41].

**Table 2:** Model predicting probability of planting all crops as a function of purchasing a forecast.

	Estimate	Std. Error	Z value	P(>|Z|)	Odds ratios (95% C.I.s)
Fixed intercept	−0.1036	0.5711	−0.181	0.8560	
Purchased a forecast	−1.3333	0.6331	−2.106	0.0352	0.26 (0.08, 0.91)
Instructions	−1.4708	1.1609	−1.267	0.2052	0.23 (0.02, 2.24)
Forecast*instructions	2.3123	1.3097	1.766	0.0775	10.1 (0.78,131.55)
Random intercept effect (player)	2.505				
Random intercept std. dev	1.583				
Inter-class correlation (complete model)	0.432				
Inter-class coefficient (adding fixed effects)	0.409				

### Research question 2

Over the games played, players bought forecasts 71.89% of the time. For players that did not buy a forecast, crop location was selected at random, and players planted crops in ways that optimized expected value only by chance. For all turns in games in which players bought a forecast, 78.03% involved planting crops at locations that maximized the expected value. This pattern did not appear to differ based on whether a player consulted game instructions (77.17%) or not (78.31%). When mistakes were made in the placement of crops, 71.59% were small mistakes that involved just switching the locations of two crops.

For the players who purchased a forecast, we model whether a player made a mistake as a function of forecast cost, the number of decision options (how many field locations were available to choose from) and the use of instructions, with a random intercept at the player-turn level. This means the variation in the analysis is at the level of the individual planting decisions occurring during a player’s turn. The results are shown in Table 3. Here we see that higher forecast cost is associated with more error. Converting into odds-ratios, we see that all else being equal, for every $1 increase in the cost of a forecast there is a 1.93% (0.34, 3.3) increase in the odds of making a mistake (or, a roughly 20% increase in the odds of making a mistake per $10). We also see that mistakes are more likely when there are more decisions to be made; for every decision that a player must make about planting a crop in a turn there is a 25.53% (14.1, 44.7) increase in the odds of making a mistake. The model results provide no evidence that using instructions influences whether a player makes a mistake about where to plant crops. Moreover, the Game # variable (measuring the effect of learning) shows an expected negative effect—such that error decreases the more one plays—but the magnitude is not inconsistent with the null hypothesis of no effect.

**Table 3:** Probability of making a mistake as a function of forecast cost and decision options available.

	Estimate	Std. Error	Z value	P(>|Z|)	Odds ratios (95% C.I.s)
Fixed intercept	−4.5728	0.7716	−5.926	<0.0001	
Forecast cost ($)	0.0193	0.0091	2.115	0.0345	1.02 (1.00, 1.04)
No. of decision options	0.2553	0.0603	4.231	<0.0001	1.29 (1.14, 1.45)
Instructions	−0.0523	0.4213	−0.118	0.9059	0.95 (0.42, 2.16)
Game #	−0.0740	0.0909	−0.814	0.4157	0.93 (1.11, 0.77)
Random intercept effect (player)	0.9406				
Random intercept std. dev.	0.9699				
Random intercept effect (Player-Game #)	1.3742				
Random intercept std. dev.	1.1723				
Inter-class correlation (complete model)	0.413				
Inter-class coefficient (adding fixed effects)	0.369				

### Research question 3

We model whether a player purchased a forecast based as a function of the net value of the forecast (NVOF) which is the difference between TVOF and the forecast cost for the player. A positive NVOF means the forecast is worth buying, and a negative NVOF means the forecast is not worth buying. Across the 711 turns played, the mean NVOF differed between for players that bought a forecast (21.51) and for players that did not (−0.02) (p < 0.001).

**Fig 2:**
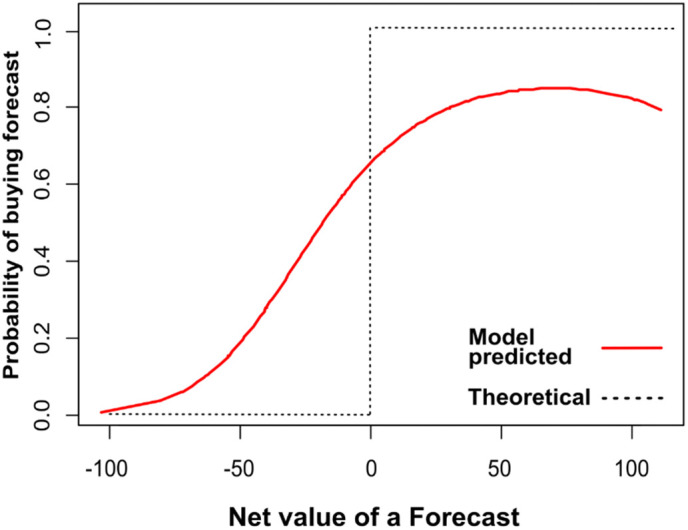
Prediction of purchasing a forecast as a function of the net value of a forecast (NVOF).

To understand this more deeply, we model the random intercept at the player-game level. The results are in Table 4. Here we see that NVOF is positively associated with purchasing a forecast. As in the previous model, instructions do not appear to have an impact on player decisions. Fig 2 shows how increasing NVOF corresponds to an increasing probability of buying a forecast. The rational expectation is that at NVOF = 0 players would be indifferent to buying a forecast, when NVOF is less than 0 they would never buy a forecast, and when NVOF is greater than 0 they would always buy a forecast. The shaded line on the graph outlines this theoretical ideal, while the red line is the modelled result from player data. We see here that at the break even point (where NVOF = 0) the model predicts that more than 60% would buy the forecast—suggesting a slight over-valuing of information on average. At the same time, the model shows that players also under-value information when NVOF is above 0.

**Table 4:** Probability of purchasing a forecast as a function of the net value of a forecast (NVOF).

	Estimate	Std. Error	Z value	P(>|Z|)	Odds ratios (95% C.I.s)
Fixed intercept	0.8876	0.3115	2.850	0.0044	
NVOF ($)	1.4950	0.2082	7.182	<0.001	4.46 (2.97, 6.71)
NVOF squared	−0.5831	0.1418	−4.113	<0.001	0.59 (0.42, 0.74)
Instructions	−0.0953	0.4919	−0.194	0.8464	0.90 (0.35, 2.38)
Random intercept effect (player-turn)	6.164				
Random intercept std. dev.	2.483				
Inter-class correlation (complete model)	0.643				
Inter-class coefficient (adding fixed effects)	0.509				

## Discussion

This study explored the impact of information on decisions made by players of an online game. Each of the three research questions concern the value of information in two senses. First, is in the sense of in-game information—a forecast that can be used by a player to augment and improve game decisions. The second sense concerns the choice to seek information about how the game is played. All players had access to information in the game, but the costs of forecasts were assigned at random for each player at the start of their first game. All players had access to game instructions at any time throughout play. The random assignment of forecasts cost means that any association between forecast cost and any decision of a player can only be explained by cost and is unconfounded by player attributes. On the other hand, the availability of instructions was not an experimental measure.

The online recruitment method, small number of participants and nascency of online games as research platforms require that we interpret all results as exploratory. Nevertheless, we discuss key findings associated with each research question below.

### Research question 1

In the first turn of play, players that did not buy a forecast planted all their crops in less than half (~40%) of games played. Under the assumption that players act rationally and use information perfectly, we expect that in turn one the players would plant their crops in all field locations—since absent any information, this is the utility maximizing decision. However, we also measured the association between buying a forecast and planting all crops and found that players who did not buy a forecast seemed more likely to plant all their crops than those who bought a forecast. This difference was small but suggests that the forecast information did what was intended for players on average—it impacted and improved the decisions players made in the game.

The random effects of the model shown in Table 2 showed that the bulk of the variation in whether or not a player planted all crops is explained by factors other than the fixed effects included in the analysis. Since these are player level effects, they can be explained by mix of personal traits and situational factors. While our research offers no definitive insight into why people made the decisions they did, these findings suggest that the incorrect decisions were a problem of understanding of either the game, the concept of expected value, or a combination of both. The game has a simple objective, and most players seem to express goal-oriented behaviour towards it. Given that the game is not competitive and players play anonymously, failures in perfect play seem mostly likely to be explained by lack of understanding rather than other rationality-limiting heuristics [42]. While people at different levels of numeracy seem to have some general understanding of expected value as a concept, there remain failures in deploying the concept properly in practice, even among highly numerate people [43].

### Research question 2

For players with forecast information, there are best locations to plant crops in a given turn. Ideally players that purchased a forecast would put their highest value crop on the field with the greatest probability of success, as this would maximize the payoff in a turn. This behaviour was observed in close to 80% of players turns. While it is hard to evaluate the precise meaning of this value, it suggests that most participants that had access to forecasts knew how to make use of them, independent of whether the participant consulted the instructions of the game. The level of numeracy required to understand the proper placement of crops is not high, but it does require that participants understand that the product of probability of success and crop value equal the payoff for the decisions they make in the game. There is ample evidence that people have poor understandings of probability and expected value in a variety of conditions [44–46], as well as evidence that the understanding of expected value appears at very young ages [47], and can depend on the context of information delivery.

More unexpectedly, among those who purchased forecast information, the price of the forecast was associated with increased error in the placement of crops. There is well-established evidence of a sunk-cost effect—that paying for information will increase the chance that it will be used [48,49]. However, existing literature does not speak to whether the cost of information increases the probability it will be used correctly, and the authors are aware of no widely known mechanism in the literature that explains this observation when using forecasts. It is possible that some participants experienced higher pressure when they paid a higher forecast cost, similar to the stress effects associated with math anxiety [50]. Given the lack of replication, the effect does not merit further speculation, but justifies further work in the future.

### Research question 3

Net value of a forecast (NVOF) is the difference between the value of a forecast and the cost of a forecast randomly assigned to players. We found that players buy forecasts when the NVOF is above 0, and that the larger the NVOF, the more likely that a player will buy a forecast. This is consistent with a generally rational valuing of information, and suggests a demand for information based on its anticipated value in a future decision. It is impossible to know whether these decisions were processed by either calculating or intuiting the value of the forecast, or guessed based on some general intuition that cheaper forecasts are worth buying. Given that way the problem was framed (in which forecasts could vary between $0 and $100) it is possible that players used a simple heuristic—such as any cost above $50 is not worth buying—that served them well, an average.

We observed that players did overvalue forecasts overall; when the NVOF = 0, players bought forecasts around 60% of the time. Overvaluing information has been studied in the context of the endowment effect (in which people are inclined to over-value something in their possession) [51].‌‌ Other research has suggested that gathering information can be a by-product of habits or norms in which acquiring more information is simply seen “a good thing to do” [52]. This may explain the overvaluing we observed. Players may have tried to reason about what the purpose of the game was, and in doing so, assumed that buying a forecast would be a good idea, whatever the numbers said. Further exploration of what explains the overvaluing may be worth consideration in future research.

Simultaneously, players undervalued forecasts when NVOF was greater than 0. The expectation is that players would buy a forecast when NVOF is greater than 0, but this only happened roughly 80% of the time. Given that the randomized forecast price for each player was the same across all turns and games they played, and that they were given no direct price framing or reference point in the game, their only option was to calculate NVOF based on the information they had, or make an intuitive judgement about fair price.

### Games in research

Participants in this research were not given financial payments, course credits or other incentives as a motivation to play. While the number of participants was not large, most played the game multiple times, garnering a modest dataset of decisions. This suggests that the activity was interesting enough to capture the attention of participants, at least for a short time. As a short casual game that could be played almost anywhere, the burden of participation was low, and this work reinforces the feasibility of using online games in primary data collection. As the literature in this domain grows, more can be learned about research gaming to design games with the right balance between information value (for the researchers) and engagement (for the participants) in order to ensure that data generated are of value.

In all of our models, a significant amount of variation was exogenous to the model fixed effects and was observed as random effects explained by latent attributes of players. We did not explore player-specific attributes, but the extant literature on decision making under uncertainty offers many possible explanations, including personal views on risk-aversion, cognitive load tolerance, levels of numeracy and mindset towards the activity. Incorporating individual attributes about participants can be captured in pre-game surveys, or within games themselves—for example, using a different game to first isolate numeracy level, and then going on to the main game. While our game was limited in scope, it helped reveal the potential of games as a platform for exploring many factors that go into decision making under conditions of uncertainty. It may also be useful platform for integrating with agent-based models in cases when agents are required to make sequential decisions [53].

### Limitations

The most significant limitation of this research concerns the participants in the study. First, there were only 54 participants. Second, participants were recruited via social media, and represented a narrow demographic range—particularly on education level and age. It follows that the results of this research are very limited in generalizability—to a well a young and well-educated population—and that they may not replicate in other populations. For these reasons, the results should be seen more as a proof-of-concept rather than a conclusive indication of decision making outside the scope of the game, or in the population in general.

Within-player effects may be exaggerated in online games when compared to other experimental platforms. Some players may be inclined to experiment or even explore game-breaking. Qualitative inspection of the data suggests that this could have been the case in some games. For example, one player tried the same strategy in multiple games. Technological issues—such as changing browser settings or other factors can contaminate data, and corruption of data in transit is also possible. All of these factors suggest that the random effects pertaining to players may be smaller using in-person or even controlled laboratory platforms. This is very likely an important trade-off to consider when using games; while it may be easier and cheaper to acquire data, the quality may at times be worse. On the other hand, the vagaries of playing games online may be the most realistic and generalizable setting for some research.

Our analysis and derivations do not consider dependence in decision making over the course of the game. In deriving NVOF, we are calculating the immediate expected benefit of the information for the current turn, thereby treating each turn as an independent decision event. A theoretically perfect player strategy could account for other factors (such as the value of saving a crop for a future turn when the probability of success on field locations seems low). Our simplified approach uses a single-turn valuation, and thus provides a simplified baseline for assessing whether players understood the fundamental value of the probability data. For the purposes of this study, which is as much as a demonstration of how a game could be used as a platform for analysis of decision making, this assumption is reasonable, but future work may consider dependence in the decision-making process. Exploring dynamic programming or Markov decision processes to model long-term play could be an important avenue for follow-up research in this area.

Some data were not collected about player activities in the game, including the time for each specific moves in the game. This would have been useful for assessing levels of attention, and more importantly, for understanding the relationship between cognitive load and error. Had we measured time per move, this could have given us an indication of the impact on the time burden on decision making. Future research will include move-specific time when possible, in order to measure or control for the impact of time, which could be a proxy for cognitive load or other factors represented by time to move.

## Conclusions

Recent advances in web frameworks, game development engines, authentication methods and most recently, generative AI has made the development of research games more feasible than ever. This increase in feasibility opens the door for researchers with little to no programming experience to design and develop games in ways that would have been prohibitively costly less than a decade ago.

The results from this research provide some insights into the value placed on information (in the form of forecasts) in a sequential decision making process. Our results are consistent with the idea of bounded rationality; players make decisions that are generally rational, but seem to be limited in their ability to make complex calculations either due to their background knowledge of expected utility, lack of interest in the game or cognitive capacity. The most interesting finding is that the more players paid for information the more likely they were to make certain types of mistakes in the game. Future work should focus on the differences between participants that were left unexplained in this analysis, and follow-up experiments should also explore whether our observation that higher information costs leads to poorer decision-making is replicable in other contexts. Other work could also explore how different ways of communicating forecasts (e.g., qualitatively vs with numbers) may influence how people making decisions.
